# Adverse events during anaesthesia at an Ethiopian referral hospital: a prospective observational study

**DOI:** 10.11604/pamj.2021.38.375.24711

**Published:** 2021-04-16

**Authors:** Joe Burgess, Gebrehiwot Asfaw, Jolene Moore

**Affiliations:** 1 National Health Service (NHS) Grampian, Aberdeen, United Kingdom,; 2 Department of Anaesthesia, Bahir Dar University, Bahir Dar, Ethiopia,; 3 School of Medicine, Medical Sciences and Nutrition, University of Aberdeen, Aberdeen, United Kingdom

**Keywords:** Anaesthesia, adverse event, critical incident

## Abstract

**Introduction:**

incident reporting systems are widely utilised within healthcare to analyse adverse events and have been shown to reduce patient harm. With data to suggest high anaesthetic-related mortality in low and middle-income countries (LMICs), such systems could allow more accurate determination of rates and types of incidents and could improve patient safety.

**Methods:**

this prospective observational study carried out over six-weeks in March to April 2019 in an Ethiopian tertiary referral hospital, included direct observations in the operating room and recording of any anaesthesia-related adverse events occurring during the perioperative period.

**Results:**

fifty surgical cases were observed during weekday daytime hours. Sixteen anaesthesia-related adverse events were observed in 12 patients, including six elective cases and six emergencies, an adverse event rate of 32% (n=16), affecting 24% (n=12) of patients. Most incidents occurred in infants less than one-year-old and those between 11-20 years (31.3%; n=5 each) and those undergoing general anaesthesia (66.7%; n=8), particularly during the induction phase (50%; n=8), the most common event being prolonged desaturation (31.3%; n=5). Most events were considered to contribute a low level of harm (56.3%; n=9). There were no intra-operative mortalities.

**Conclusion:**

this study presents evidence of a higher rate of adverse events during anaesthesia at a tertiary referral hospital in Ethiopia, than reported in current literature from LMICs. There is potential for large volume data to be produced and learnt from with a reporting system in place in this setting. The most common event was desaturation detected by pulse oximetry, particularly in paediatric surgery.

## Introduction

It has been estimated that in 2010 almost a third of all deaths worldwide were caused by conditions requiring surgical care [1]; surpassing deaths from human immunodeficiency virus (HIV), tuberculosis and malaria combined [2]. A report by The Lancet Commission on Global Surgery published in 2015 highlighted the need for universal access to safe, affordable surgical and anaesthetic care [3]. Perioperative mortality has been used as an indicator of surgical and anaesthetic safety [4]; although perioperative mortality has declined over the past 50 years, low and middle income countries (LMICs) have a two to four fold increased risk of perioperative mortality [5]. Surgical patients in Africa have significantly increased mortality despite having a lower risk profile and undergoing less-complex surgeries [6]. Anaesthesia related mortality in sub-Saharan African countries is higher than in high income countries [7-10] with improvements in anaesthesia services described as a priority in global health [11]. The variability of conditions in which anaesthesia is provided in LMICs is vast; the training background of anesthetic providers can differ [12], with shortages of physician anaesthesia providers; the World Federation of Societies of Anaesthesiologists workforce survey found a 90-fold difference between the average physician anaesthesia provider workforce density in high income countries (HICs) when compared to low-income countries [13].

A critical incident in anaesthesia is defined as any untoward and preventable mishap associated with the administration of general or regional anaesthesia and which leads to or could have led to an undesirable patient outcome. Patients' safety can thus be improved by learning from reported critical incidents. Critical incident reporting, introduced in the second world war to improve safety among pilots, was implemented as a method of investigating preventable incidents in anaesthesia in 1978 [14]. Incident reporting systems are now widely utilised by anaesthetic departments in HICs to highlight, discuss and learn from adverse incidents [15], but similar data from LMICs is lacking [16]. Reviewing adverse events, critical incidents and near misses in anaesthesia has been shown to decrease their recurrence and therefore decrease patient harm [17].

The aims of this study were to investigate the incidence and types of adverse event during anaesthesia occurring in a major referral hospital in Ethiopia. Ethiopia is the second most populous nation in Africa. Its large land mass contributes to problems with access to surgery resulting in late presentations and high levels of mortality and morbidity [18]. This study reports on perioperative adverse anaesthetic events observed in patients undergoing surgery at one of the country´s major referral hospitals.

## Methods

**Study design and setting:** Felege Hiwot Referral Hospital (FHRH) is a 500 bed tertiary referral hospital located in the Amhara region of Northern Ethiopia. FHRH serves a population of around 7 million and provides surgical services in ear, nose and throat (ENT), maxillofacial, urology, general (including emergency neurosurgery), obstetrics and gynaecology, ophthalmology, orthopaedic and paediatric surgery in its 11 operating rooms. At the time of the study, anaesthesia was provided by 14 non-physician anaesthesia providers (NPAPs). This study included observations of surgical cases at the facility over a six week period, in order to ascertain the incidence and type of adverse events occurring during anaesthesia. Direct observations were included to overcome problems related to standard methods of reporting, such as reporting inconsistencies and attempt to obtain a more accurate picture of anaesthetic adverse events occurring in this setting.

An adverse event was defined according to a systems-based approach. Airway-related events included difficult and failed intubation, laryngospasm, regurgitation with aspiration and the “can´t intubate, can´t ventilate” scenario. Respiratory events included bronchospasm and desaturation/hypoxia, defined as a pulse oxygen saturation of less than 90% for more than 3 minutes. Cardiovascular events included significant hypotension, defined as a drop in blood pressure of more than 30% below baseline or systolic blood pressure below 70 mmHg for over 10 minutes and intraoperative cardiac arrest. Events relating to regional anaesthesia complications were included - failed spinal anaesthesia, high spinal block, defined as a block above T4 level with cardiorespiratory compromise and total spinal block (high spinal criteria plus loss of consciousness). Other events included were medication and equipment related - anaphylaxis, drug reaction or drug error (wrong drug, dose or route), equipment fault, loss of oxygen supply, failure of anaesthetic machine or ventilator and critical loss of power supply.

Where an adverse event occurred, the details of the case and event were recorded, including the phase of anaesthesia in which the event occurred (induction, maintenance or emergence). The severity (level of harm) of each adverse event was defined through assessment of required interventions and post-operative condition of the patient. A five-point system was used to grade level of harm, with no harm categorised as “none or near miss”, short-term harm requiring extra monitoring or a minor intervention categorised as 'low´, short-term harm requiring an intervention or medical treatment categorised as “medium” and permanent or long-term harm categorised as “severe”. The fifth and final grade was death.

**Study population:** this prospective observational study involved direct perioperative observations of patients undergoing anaesthesia for surgery during weekday daytime hours (defined as 08: 00 to 16: 00, Monday to Friday) over a six-week period in March to April 2019. Cases were observed in the main (general, urology, paediatric, ENT) and orthopaedic operating suites therefore obstetrics, gynaecology and ophthalmology were excluded. Elective and emergency surgical cases were included, as were both general and regional anaesthesia techniques. Patients of all ages were included.

**Data collection:** prior to commencing observations, a paper-based checklist was created to record case demographics including age, gender, American Society of Anesthesiologists (ASA) grade, emergency or elective case, anaesthetic technique, presence of different monitoring modalities during anaesthesia and surgery and any of the listed anaesthesia related adverse events that occurred perioperatively. Observations were undertaken by Joe Burgess (JB), with the checklist completed for each case. Where adverse events occurred, a description of the scenario and event were recorded as free-text. Where there was ambiguity over the cause or type of event, the opinion of the anaesthesia provider was sought and recorded separately on the checklist, following management of the event once the patient was stable and/or the case completed, and the case was later analysed by JB and Jolene Moore (JM). Postoperative follow-up of patients was conducted where an event occurred. The checklist was designed to capture events and not to establish root cause analysis.

**Data analysis:** data was transferred to electronic format and analysed using Microsoft Excel and is presented as descriptive statistics, including absolute numbers and percentage proportions.

**Ethical considerations:** ethical approval was obtained from the institutional review board of Bahir Dar University (protocol number 00250). Consent was obtained from patients and anaesthesia providers to observe in the operating room during anaesthesia and surgery.

## Results

**Case demographics:** a total of 50 cases were observed over the six week study period, 56% (n=28) were elective surgical cases, with the remainder emergencies. Anaesthesia mode included 60% (n=30) general anaesthesia, 56% (n=28) with airway intervention and 4% (n=2) without and the remaining 40% (n=20) under spinal anaesthesia. Patients were majority female (68%; n=34), ASA class I (66%; n=33) or II (28%; n=14) and 21-60 years of age (48%; n=24). Infants under one year constituted 8% (n=4) of cases, children aged 1-10 years 14% (n=7), 11-20 years 18% (n=9) and the remaining 12% (n=6) over 60 years. Surgical specialty mix included general (38%; n=19), orthopaedic (40%; n=40), urology (14%; n=7), and ENT/maxillofacial (8%; n=4).

**Intraoperative monitoring:** all patients had continuous heart rate and oxygen saturations monitored by pulse oximetry and all but four infants (92%; n=46) had non-invasive blood pressure measurement at least every five minutes. Precordial stethoscopes were in use in the four infants. End tidal carbon dioxide monitoring was only available in one of the seven theatres and therefore only observed in 6% (n=3) cases (all cases carried out in that theatre). Temperature monitoring did not occur in any cases.

**Adverse events:** according to the criteria set out, there were 16 adverse events observed in 12 patients, an incidence of 32%, with an even mix of elective (n=6) and emergency (n=6) cases. The distribution of adverse events was 66.7% (n=8) male and 33.3% (n=4) female. Most incidents occurred in infants less than one year (31.3%; n=5; 3 patients) and those aged eleven to twenty years (31.3%; n=5; 3 patients), with the remaining 6 incidents spread across the remaining age ranges. Two thirds (66.6%; n=8) of patients were ASA class II. Three separate events were observed in two patients, one of whom was ASA class IV.

Two thirds of patients were undergoing general anaesthesia (66.7%; n=8). Most events occurred during induction (50%; n=8) followed by maintenance (31.3%; n=5) and emergence from anaesthesia (18.8%; n=3). The commonest category of event was respiratory, specifically desaturation/hypoxia, 31.3% (n=5) of all incidents observed. Three of the five desaturation events occurred in infants less than one year. The second most common event was failed spinal. There were no drug related events. There were multiple power outages although these did not result in any adverse events as anaesthesia machines all had battery life to rely upon temporarily.

Of the 16 incidents, three quarters resulted in either no harm or a low level of harm. The events observed did not lead to any severe or permanent harm or intra-operative deaths. Adverse events are summarised in Table 1 and details of cases with observed adverse events are illustrated in Table 2.

**Table 1:** adverse events during anaesthesia

Category and adverse event	Percent of cases (n)
Airway	25% (4)
Difficult intubation (>3 attempts)	12.5% (2)
Failed intubation	0
Can't intubate, can't ventilate scenario	0
Laryngospasm	6.3% (1)
Regurgitation with aspiration	6.3% (1)
Respiratory	31.3% (5)
Bronchospasm	0
Desaturation/hypoxia (all categories)	31.3% (5)
Desaturation/hypoxia (SpO2 <90% for >3mins)	18.8% (3)
Desaturation/hypoxia (SpO2 <85% for >3mins)	12.5% (2)
Circulatory	12.5% (2)
Hypotension (>30% below baseline for >10 mins or SBP <70mmHg for >10 mins)	12.5% (2)
Intraoperative cardiac arrest	0
Spinal anaesthesia related	25% (4)
Failed (>2 attempts with no block or inadequate block requiring supplementation)	18.8% (3)
High (associated with cardiorespiratory compromise + block >T4 (if checked))	6.3% (1)
Total (high spinal + loss of consciousness)	0
Drug related	0
Anaphylaxis	0
Other adverse drug reaction	0
Drug error (wrong drug/dose/route)	0
Equipment	6.3% (1)
Equipment fault	6.3 (1)
Loss of oxygen supply	0
Failure of anaesthetic machine or ventilator	0
Critical loss of power	0
Level of harm	
None or near miss: no harm occurred	18.8% (3)
Low: short term harm requiring extra monitoring or minor intervention	56.3% (9)
Medium: short term harm requiring intervention or medical treatment	25% (4)
Severe: permanent or long-term harm	0
Death	0

**Table 2:** summary of adverse events

Age range	Sex	ASA	Urgency	Specialty	Anaesthesia	Event	No. of events	Phase	Level of harm
<1	M	2	Emergency	General	General	Aspiration hypoxia	3	Induction emergence	Medium
>60	M	2	Emergency	Urology	General	Hypotension	1	Maintenance	Low
21-60	M	1	Emergency	Urology	Spinal	Failed spinal	1	Maintenance	Low
11-20	F	4	Emergency	General	General	Hypoxia equipment fault	3	Induction emergence	Low Med
<1	M	2	Elective	General	General	Hypoxia	1	Induction	None
<1	M	2	Emergency	General	General	Difficult intubation	1	Induction	Low
21-60	M	1	Emergency	General	General	Hypotension	1	Maintenance	Low
>60	M	2	Elective	General	Spinal	High spinal	1	Maintenance	Low
21-60	F	2	Elective	General	General	Difficult intubation	1	Induction	None
1-10	M	2	Elective	Ortho	General	Laryngospasm	1	Emergence	None
11-20	M	2	Elective	Ortho	Spinal	Failed spinal	1	Maintenance	Low
11-20	F	1	Elective	Ortho	Spinal	Failed spinal	1	Induction	Low

Ortho: orthopaedic surgery

## Discussion

The reporting of critical incidents and adverse events is of vital importance to the specialty of anaesthesia; discussion of these incidents can inform policies or training programmes to prevent recurrence and ultimately improve patient care [15,16]. The incidence of adverse events observed in this study (32%) is considerably higher than reported elsewhere with rates as low as 0.92% in Zimbabwe [19] and 6.1% in Nigeria [20]. Most critical incidents in this study occurred during induction, which is often seen as an ‘incident rich´ phase [21]. Induction events contribute a significant proportion of events in other studies but are often eclipsed by maintenance phase events [22,23].

A high proportion of adverse events in this study occurred in infants less than one-year old. Our study is in keeping with reports of the commonest reported events being respiratory complications [21,24-26], with desaturation events the most frequently occurring events in infants. Anatomical and physiological differences between adults and children create challenges to anaesthesia providers with children demonstrating tendency to more rapid desaturation. Sub-Saharan countries are home to a large proportion of children, of which it has been estimated up to 85% will need treatment for a surgical condition by the time they reach fifteen [27]. Paediatric anaesthetic related mortality in LMICs is reported as 2.4-3.3 per 10,000 anaesthetics [28,29], higher than reported HIC rates of less than 1 per 10,000 [30-32]. The high demand for paediatric surgery added to higher anaesthetic related mortality and adverse events highlights the need for focus on safety of paediatric anaesthesia services in LMICs.

Failed or inadequate spinal anaesthesia represents a significant proportion of adverse events observed in this study. The levels of harm were low for these cases. A contributing factor may be patient positioning during spinal anaesthesia; in this study the patients sat on the operating table with legs flat in front, a method associated with less successful first attempts than sitting with legs on a side stool [33]. Adverse and critical incident rates in current literature largely rely on retrospective analysis of records and voluntary submission of incident forms, which can result in incomplete data sets. One study of compliance with filling these forms found only 30% of incidents were being reported [34]. Variations in rates could also be explained by differences in individual perceptions of what qualifies as an adverse event or critical incident and the potential for underreporting due to fear of consequences, lack of understanding of the possible learning implications and variations in “no blame” culture. The current study reports on directly observed events.

A possible reason for the high incidence in this study is a potentially lower threshold for reporting, including adverse as well as ‘critical´ incidents. Of the 16 events in this study, 12 were judged to carry no or low severity/harm and similar events may be judged as not worthy of reporting in other settings or by other groups. For example, hypotension or hypoxia events may not be reported unless resulting in harm or related to significant cause e.g. embolic events. There are also other factors to consider, for example two patients had multiple events, which may in some systems be reported as one event combined. This study also did not consider other variables e.g. first attempts at intubation or spinal anaesthesia carried out by students. This exposure to anaesthesia is essential for the students´ learning but could be a factor increasing the occurrence of adverse events.

This study has some limitations; the study observed cases in daytime only and a proportion of events may occur out of hours, nor did this study analyse events in-depth considering pre-morbid condition, severity of surgery or potential contribution of surgical-related causes e.g. hypotension related to pre-existing sepsis or intraoperative haemorrhage. In order to fully investigate and learn from adverse events, a more in-depth analysis of cause is beneficial. This study may also be impacted by language barrier between observer and anaesthesia provider causing misunderstanding of events. It is also important to consider the low case numbers in this study due to its prospective nature.

## Conclusion

Critical incident and adverse event reporting systems can enable monitoring, analysis and discussion which may lead to training or policy interventions. This study conducted in Ethiopia, at a facility where no such reporting system exists, revealed a higher incidence of adverse events than reported in current literature pertaining to adverse events during anaesthesia. The most common anaesthetic related event was desaturation detected by pulse oximetry, particularly in paediatric surgery. In order to improve anaesthesia and surgical safety, surgical morbidity and mortality surveillance is necessary to identify preventable causes and potential interventions. The use of direct observations of adverse events in this study suggests an accurate reporting system could have potential for large volume data to be produced and learnt from in this facility.

### What is known about this topic

- Anaesthesia related mortality in sub-Saharan African countries is higher than in high-income countries;
- Reported rates of anaesthesia related adverse events vary;
- Reviewing adverse events can decrease their recurrence and decrease patient harm.

### What this study adds

- Anaesthesia related adverse events observed at a major referral hospital in Ethiopia are high;
- Paediatric surgical cases are at increased risk.
